# Chemical Composition, Modulatory Bacterial Resistance and Antimicrobial Activity of Essential Oil the *Hyptis martiusii* Benth by Direct and Gaseous Contact

**DOI:** 10.17795/jjnpp-13521

**Published:** 2014-06-29

**Authors:** Allan Demetrius Leite de Oliveira, Fabiola Fernandes Galvao Rodrigue, Henrique Douglas Melo Coutinho, Jose Galberto Martins da Costa, Irwin Rose Alencar de Menezes

**Affiliations:** 1 Department of Biological Chemistry, Laboratory of Pharmacology and Molecular Chemistry, Regional University of Cariri-URCA, Crato-CE, Brazil; 2 Faculty of Leao Sampaio, Lagoa Seca Campus, Juazeiro do Norte-CE, Brazil; 3 Department of Biological Chemistry, Laboratory of Natural Product Research, Regional University of Cariri-URCA, Crato-CE, Brazil; 4 Department of Biological Chemistry, Laboratory of Microbiology and Molecular Biology, Regional University of Cariri-URCA, Crato-CE, Brazil

**Keywords:** Essential Oils, *Hyptis martiusii*, Antimicrobial Resistance, Aminoglycoside

## Abstract

**Background:**

Several studies have shown that species of the genus *Hyptis*, have promising antimicrobial and antifungal effects.

**Objectives:**

Identify of chemical constituents of essential oil from leaves of *Hyptis martiusii* and evaluate its effect against bacterial strains by direct and gaseous contact.

**Materials and Methods:**

Essential oil was extracted from leaves of *Hyptis martiusii* Benth using hydro-distillation, and its composition was determined using gas chromatography–mass spectrometry (GC-MS). Chemical analysis showed that there was a predominance of sesquiterpenes. The leaf essential oil was screened for its minimal inhibitory concentration and modulatory effect of aminoglycoside by the direct (MIC) and gaseous (MID) micro-dilution assays for various pathogenic microorganisms. The essential oil remarkably inhibited the growth of all of the tested bacteria (MIC < 512 μg/mL) except *S. aureus* (SA358) multidrug resistant (MRSA) by direct contact.

**Results:**

Twenty-four compounds representing 92.13% of the essential oil of leaves were characterized; δ -3-carene (6.88%), 1, 8-cineole (7.01%), trans-caryophyllene (9.21%), Cariophyllene oxide (7.47%) and bicyclogermacrene (10.61%) were found as the major components. Modulatory aminoglycoside effect, by direct contact, was showed antagonistic relationship with antimicrobial activity. The gaseous component of the oil inhibited the bacterial growth of all of the tested bacteria in 50% and 25% of oil concentration and demonstrated synergistic interactions can be attributed to the constituting the oil compounds.

**Conclusions:**

These results show that this oil influences the activity of the antibiotic and may be used as an adjuvant in the antibiotic therapy of respiratory tract bacterial pathogens.

## 1. Background

Microorganisms are responsible for a vast majority of pathologies presented to man, and bacteria are a huge source of pathogens as they are susceptible to genetic modification, making them tougher to fight in most cases. Some groups of bacteria, especially *Escherichia coli* and *Staphylococcus aureus* are among the most representatives associated with gastrointestinal tract infections and skin diseases. According to Fagundes-Neto and Scaletsky (1). Diarrheal disease is a major cause of death among children younger than 5 years in low-income populations of developing countries, and the same with the primary causative *E. coli*. This is still correct for high rates of nosocomial infections, being classified amongst the four most prevalent microorganism, as well as *Pseudomonas aeruginosa*, as an opportunistic microorganism. *E. coli* is often isolated from urinary tract infections; the intensity of infection depends on the virulence of the contaminating strain and host susceptibility (2).* S. aureus* is the most common etiologic agent in inflammatory infections such as abscess, myocarditis, endocarditis, pneumonia, meningitis and bacterial arthritis (3). This bacteria is also described as a major nosocomial agents, where several factors are associated with their resistance in hospital settings, as well as the presence of urinary catheter, prior antibiotic use, use of central venous catheters, mucositis among others, which explains the high rate of morbidity and mortality from *S. aureus* in intensive care units (ICUs) (4, 5). The use of natural products as antimicrobial agents is a widespread practice and it can often yield results satisfactory, hence its wide use (6). However, it is worth noting that these natural products can also be sources of rich concentrations of toxic substances, making them harmful to humans. Some species of lamiaceae seem to have chemicals capable of inhibiting bacterial growth. This family includes about 220 genera and over 4000 species. A small number of papers describing the biological activities of the species *Hyptis martiusii* as larvicidal and insecticidal activities (7, 8), antimicrobial (9), genotoxic, phototoxic and cytotoxic activity (10-12), antiproliferative (13) and antiulcerogenic (14). However, it is known that research has been carried out with the family lamiaceae and even species of the genus *Hyptis*, where several of the show as a promising antimicrobial and antifungal (8, 15-25).

## 2. Objectives

This study was carried out in order to achieve the identification of chemical constituents of essential oil from leaves of *Hyptis martiusii* and evaluate its effect against strains of *E. coli* and *S. aureus* (ATCC and clinical origin of multidrug-resistant), *B. cereus* and *P. aeruginosa* strains ATCC by direct and gaseous contact and modulatory effect in resistance bacterial.

## 3. Materials and Methods

### 3.1. Bacterial Material

The test microorganisms included two Gram-positive bacteria, *S. aureus* ATCC 12692 and clinical isolates *S. aureus* 358 (SA358), and *Bacillus cereus* ATCC and two gram-negative bacteria, *E. coli* ATCC 25922 and clinical isolates *E. coli* 27 (EC27) and *P. aeruginosa* (ATCC). The bacterial strains utilized where the clinical isolates were identified by standard procedure with the resistance profile described in Table 1. All strains were maintained on slants with heart infusion agar (HIA, Difco Laboratories Ltda.). Before the assay, the cells were grown overnight at 37°C in brain heart infusion (BHI) broth (BHI, Difco Laboratories Ltda).

**Table 1.** Chemical Compostion of Essential Oil From *Hyptis martiusii* Benth_a_

Constituents	Retention Time, min	Compositions, %	KI
**δ-2-carene**	9.6	6.8	1002
**1, 8-cineole**	10.6	7.0	1031
**Trans-caryophyllene**	30.9	9.2	1409
**Aromadendrene**	31.8	2.7	1441
**α-humulene**	32.7	2.2	1455
**Germacrene-D**	34.0	3.8	1482
**Ledene**	34.5	5.4	1485
**Valencen**	34.8	1.5	1496
**Torreiol**	42,8	2.6	1645
**α-eudesmol**	43.4	3.7	1654
**Bicyclogermacrene**	35.8	10.6	1500
**β- guaiene**	36.0	2.4	1503
**δ-cadinene**	36.8	3.1	1523
**Epiglobulol**	37.0	2.3	1564
**Cariophyllene oxide**	39.0	7.4	1583
**Globulol**	39.2	2.1	1585
**δ- guaiane**	39.6	2.4	1596
**Guaiol**	39.8	3.3	1601
**10-epi-α-eudesmol**	41.5	1.3	1624
**Terpinolene**	12.9	1.6	1089
**Camphor**	16.3	2.6	1146
**Total**		84.0	

^a^ Abbreviation: KI, Kovats retention index

### 3.2. Plant Material and Isolation of Essential Oils

For the extraction of essential oils from the leaves, plants were collected locally from Chapada do Araripe in Crato City situated in the south of the State of Ceara, Brazil. A voucher specimen has been deposited with the number 4610 at Herbarium “Dardano de Andrade Lima” of Universidade Regional do Cariri - URCA. The air-dried, powdered and mature flowering twigs (274 g) of *Hyptis martiusii* were hydro-distilled in Clevenger-type apparatus for 3 hours. The isolated fractions of plant parts exhibited two distinct layers-an upper oily layer and the lower aqueous layer. Both the layers were extracted with diethyl ether, separated and the ethereal layer was dried over anhydrous sodium sulphate. The essential oils were stored at 4°C in a clean amber glass bottle until used.

### 3.3. Gas Chromatography–Mass Spectrometry Analysis

**Figure 1.**
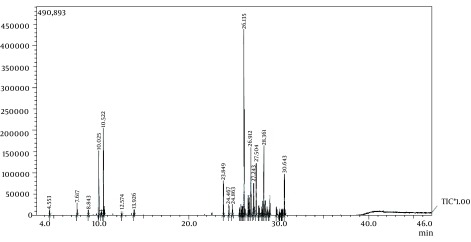
Chromatogram of the Essential oil of *H. martiusii*

Gas chromatography – mass spectrometry analysis of the volatile oil was carried out on a GC/MS (SHIMADZU) equipped with a QP5050A detector and DB-5HT capillary column 30 m 0.25 mm i.d. film thickness 0.25 Lm. The oven temperature was held at 60°C for 6 minutes, programmed at 5°C/min to 270°C and then held for 10 minutes. The carrier gas was helium at a flow-rate of 1.7 mL/min (split mode). The injector and detector temperatures were 270 and 290°C. The quadruple mass spectrometer was scanned over the 30-400 amu range at 1 scan/s with an ionizing voltage of 70 eV. Retention indices were calculated using cochromatographed standard hydrocarbons. The individual compounds were identify by MS and their identity was confirmed by comparing their retention indices relatives to C8-C32 n-alkanes and by comparing their mass spectra and retention times with those of authentic samples or with data already available in the Wiley 229 library and literature (Figure 1) (26).

### 3.4. Antibacterial Test (Minimal Inhibitory Concentration)

Minimal inhibitory concentration (MIC) of the essential oil from *H. martiusii* was determined in a micro-dilution assay, recommended by NCCLS M7-A6 (27). The assay was carried out with four bacterial species: *S. aureus* (ATCC 6538) and *E. coli* (ATCC 25922) and two bacterial *S. aureus* (SA358) and *E. coli* (EC27) clinical isolate presenting multi-resistance for diverse antibiotics (Table 2). Brain- Heart Infusion (BHI 3.8%) broth was used for bacterial growth (24 hours, 35 ± 2°C). The inoculum was an overnight culture of each bacterial species in BHI broth diluted in the same media to a final concentration of approximately 10^8^ colony forming unit-CFU/mL (0.5 nephelometric turbidity units-McFarland scale). Afterwards, the suspension was diluted to 10^6^ CFU/ml in 10% BHI. Hundred milliliters of each dilution was distributed in 96-well plates plus essential oil in different concentrations (1024 to 1 μg/mL), achieving 5 × 10^5^ UFC/mL as the final concentration of the inoculum. The essential oil of the *Hyptis martiusii* was dissolved in distilled water and dimethyl sulfoxide (DMSO) to a concentration of 10^3^ mg/mL. Further serial dilutions were performed by addition of BHI broth to reach a final concentration in the range was 1024, 512, 256, 128, 64, 32, 16, 8, 4, 2 and 1 μg/mL. All experiments were performed in triplicate, and the micro-dilution trays were incubated at 35 ± 2°C for 24 hours. Antibacterial activity was detected using a colorimetric method by adding 25 µL of resauzurin, staining (0.01%) aqueous solution in each well at the end of the incubation period. The minimal inhibitory concentration (MIC) was defined as the lowest essential oil concentration able to inhibit the bacterial growth, as indicated by resauzurin staining (bacterial dead cells are not able to change the staining color when visually observed-blue to red).

**Table 2.** Clinical Isolates Bacterial Antibiotic Resistance ^a^

Bacteria	Source	Antibiotic Resistance
***Escherichia coli*** ** EC27**	surgical wound	Ast, Ax, Amp, Ami, Amox, Ca, Cfc, Cf, Caz, Cip, Clo, Im, Can, Szt, Tet, Tob
***Staphylococcus aureus*** ** SA358**	surgical wound	Oxa, Gen, Tob, Ami, Can, Neo, Para, But, Sis, Net

^a^ Abbreviation: Ast, aztreonam; Ax, amoxicillin; Amp, ampicillin; Ami, amikacin; Amox, amoxicillin; Ca, cefadroxil; Cfc, cefaclor; Cf, cefalotin; Caz, ceftazidime; Cip, ciprofloxacin; Chlo, chloramphenicol; Im, imipenem; Kan, kanamycin; Szt, sulfametim; Tet, tetracyclin; Tob, tobramycin; Oxa, oxacillin; Gen, gentamicin; Neo, neomycin; Para,paramomycin; But, butirosin; Sis, sisomicin; Net, netilmicin.

### 3.5. Modulation of Antibiotic Activity by Direct Contact

The MICs were recorded as the lowest concentration for growth inhibition. The Minimal bactericidal concentration (MBC) was determined inoculating samples from non-growth wells on plates with BHI agar. The isolate clinical bacteria strains EC27 and SA358 were assayed with four different aminoglycosides with final concentrations of 1024, 512, 256, 128, 64, 32, 16, 8, 4, 2 and 1 μg/mL. All plates were incubated aerobically for 24 hours at 37ºC. Antibacterial activity was detected using a colorimetric method by adding 25 µL of resauzurin, staining (0.01%) aqueous solution in each well at the end of the incubation period. The MBCs were recorded as the lowest concentration without growth. All antimicrobial assays were performed twice and the results were expressed as average of the tree repetitions. For the evaluation of the modulator effect of antibiotic resistance, MICs of the antibiotics were determined in the presence of the essential oil solutions (concentration 10 μg/mL) and in the presence of different aminoglycosides (concentration 16 μg/mL) at sub-inhibitory concentrations. The plates were incubated for 24 hours at 37°C. Antibacterial activity was detected using a colorimetric method by adding 25 µL of resauzurin, staining (0.01%) aqueous solution in each well at the end of the incubation period.

### 3.6. Determination of Minimal Inhibitory Dose (MID) of Essential Oil by Gaseous Contact

Antibacterial activity of the essential oil from *Hyptis martiusii* was assayed using gaseous contact. An amount of 50 μg of oil was dissolved in 50 μL of DMSO (1:1). A twofold dilution series of this essential oil solution was prepared: 50, 25, 12.5 and 6.25 μg of oil. Petri dishes with nutrient agar (Difco) were inoculated with 10^5^ CFU/mL by the spread plate method. A volume of 100 μL of each dilution was placed inside the upper part of Petri dish. The plates were incubated at 37°C for 24 hours. The minimal inhibitory dose (MID) was defined as the minimal inhibitory dose per unit space required to suppress the growth of microorganism in a closed system. The MID values were expressed as weight per unit volume (mg/L air), where the solution with 50 μg equals 1 mg/L air (28).

### 3.7. Antibiotic Modifying Activity by Gaseous Contact

The antibiotic modifying activity of the gaseous component was determined using the same method, but only the solution with a total of 50 μg of oil was used. In these plates, antibiotics disks with gentamicin, amikacin and tobramycin were used to determine changes in the inhibition zone diameter of *S. aureus* ATCC 12692, *S. aureus* 358 (SA358), *B. cereus* ATCC 33018, *E. coli* ATCC 25922, *E. coli* 27 (EC27) and *P. aeruginosa* ATCC 15442. Plates without the essential oil and with DMSO alone were used as control.

### 3.8. Statistical Analysis

The gaseous contact results was made in triplicate and expressed as mean ± SEM. For statistical analysis, ANOVA followed by Tukey's post hoc test, as appropriate, were used. A P < 0.05 was considered statistically significant.

## 4. Results

### 4.1. Chemical Composition of the Essential Oil

*H. martiusii* is an important medicinal plant used local community for the curing of many diseases. The volatile oil of the mature flowering twigs of *H. martiusii* (EOHm) was obtained by conventional hydro-distillation method using a Clevenger-type apparatus and the yield of the oil was found to be in 0.78% (w/w), based on dry weight. The essential oil produced by this plant is a complex mixture of terpenes, sesquiterpenes, their oxygenated derivatives and other aromatic compounds. Forty one components (Table 1) accounting for 84% of the essential oil of *H. martiusii* (EOHm), retention time, relative retention index (experimental and from literature), and percentage are reported were identified. The main components identified were bicyclogermacrene (10, 60%) and bicyclogermacrene (9.2%), 1,8-cineole (7.0%), δ-2-careen (6.8%) and ledene (5.4%) represented the most abundant compounds as main compounds along with 21 other minor constituents.

### 4.2. Antimicrobial Activity

The in vitro antimicrobial activity of the essential oil of *H. martiusii* (EOHm) shows the oil were able to inhibit bacterial growth (antibacterial effect) in *E. coli*, *B. cereus*, *P. aeruginosa* and *S. aureus*, ATCC strains and *E. coli* and *S. aureus* multidrug-resistant (Table 2) was showed in Table 3. The results of this study showed antibacterial activity to the standard strain (ATCC) of all bacteria with MIC of 512 μg/mL except *B. cereus* where MIC 256 μg/mL and *S. aureus* SA358 where MIC ≥ 1024 μg/mL. The results of the MICs were more effective against *E. coli* multidrug resistant with MIC of 64 μg/mL that the ATCC2592 512 µg/mL.

**Table 3.** Minimal Inhibitory Concentration Values (μg/mL) of Aminoglycosides and EOHm

Bacterial	*E. coli*, (EC27)	*E. coli*, (ATCC2592)	*S. aureus*, (SA358)	*S. aureus*, (ATCC1269)	*B. cereu, *(ATCC33018)	*P. aeruginosa*, (ATCC15442)
**EOHm**	64	512	≥ 1024	512	256	512
**Amikacin**	64	256	256	256	128	128
**Neomycin**	32	256	128	128	256	64
**Gentamicin**	16	64	64	64	256	64
**Kanamycin**	16	256	128	64	256	128

### 4.3. Modulatory Bacterial Resistance Effect

The modulator effect with aminoglycoside antibiotics (amikacin-AMI, kanamycin-KAN, neomycin-NEO and gentamycin-GENT or tobramycin-TOBRA), was designed to evaluate a possible interaction between the product and the natural antibiotics in order to check the activity synergistic or antagonistic. The modulatory activity of aminoglycoside by direct contact was showed witch antagonist effect in *E. coli* ATCC25922 (MIC 16 for 64 μg/mL) and *S. aureus* ATCC12692 (MIC 16 for 128 μg/mL) in presence of neomycin, *P. aeruginosa* ATCC15442 (MIC 14 for 64 μg/mL) in presence of amikacin and *S. aureus* 358 in presence of neomycin (16 for 512 μg/mL), amikacin (16 for 64μg/mL) and kanamycin (16 for 128 μg/mL) (Table 4).

**Table 4.** Minimal Inhibitory Concentration Values (μg/mL) Combination of Aminoglycosides (MIC/8) in the Absence and Presence of 8 μg/mL of EOHm ^a^

Bacteria	AMI		GENT		NEO		KAN	
	OE	CONT	OE	CONT	OE	CONT	OE	CONT
***E. coli*** ** ATCC25922**	32	32	64	64	64	16	64	64
***E. coli*** ** 27**	32	32	64	64	16	16	32	32
***S. aureus*** ** ATCC12692**	128	128	64	64	128	16	128	128
***S. aureus*** ** 358**	256	4	128	64	512	16	128	16
***P. aeruginosa*** ** ATCC15442**	64	16	512	512	32	32	128	128
***B. cereus*** ** ATCC33018**	32	32	64	64	16	16	64	64

^a^ Abbreviations: AMI, amikacin; GENT, gentamycin; NEO, neomycin; KAN, kanamycin; OE, essencial oil; CONT, negative control (aminoglycoside only).

Differently of the results presented by the antimicrobial activity by direct contact, the actions of volatiles compounds present in essential oil of *Hyptis martiusii* Benth w demonstrated a significant synergism front to different bacteria. The results obtained in the antimicrobial assays are presented in Table 5. This results show a broad spectrum of a remarkable activity against all tested strains in presence of EOHm at 50 and 25% of concentrations and the diameters of growth inhibition zones ranged from 14 to 60 mm. 

Furthermore, significant reductions in bacterial growth were obtained with *S. aureus* ATCC12692 increment of inhibition 328.9% and 171.7% for amikacin in concentration of 50% and 25% of EOHm, respectively. Other synergistic effects were observed against *E. coli* ATCC25922 with increment of inhibition 76.5% and 64.7% for tobramycin in gaseous contact of essential oil at concentration of 50% and 25% and *E. coli* 27 (multiresistant) an increment of inhibition 82.4 and 80% for amikacin and tobramycin and *B. cereus* ATCC 33018 show increment of inhibition arrived to 77.8% when in contact with the amikacin respectively, all in the concentration of 50%.

**Table 5.** Modification of the Antibiotic Activity of the Volatile Compounds of *H. martiusii* Essential oil by Gaseous Contact ^a, b, c^

Antibiotic	N. TRA	DMSO	EOHm, 50%	EOHm, 25%	EOHm, 12%	EOHm, 6%
***S. aureus*, ATCC12692**						
Control GENT	16.3 ± 0.6	16.7 ± 0.6	60.0 ± 0.5 ^a^	60.1 ± 0.6 ^a^	16.3 ± 0.8	16.3 ± 0.5
Increase, %	-	-	268.1	268.7	-	-
Control AMI	17.3 ± 0.6	17.3 ± 0.6	74.2 ± 0.0 ^a^	47.0 ± 0.5 ^a^	17.3 ± 0.7	17.3 ± 0.0
Increase, %	-	-	328.9	171.7	-	-
Control TOBRA	15 ± 0.7	15.3 ± 0.7	57 ± 0.3 ^a^	34±0.0 ^a^	25 ± 0.3 ^a^	18 ± 0.0 ^a^
Increase, %	-	-	280	126.7	66.7	20
***P. aeruginosa*, ATCC15442**						
Control GENT	14.3 ± 0.6	14 ± 0.0	17 ± 0.7 ^a^	14.3 ± 0.4	14.3 ± 0.8	14.3±0.0
Increase, %	-	-	18.9	-	-	-
Control AMI	15 ± 0.0	15.3 ± 0.6	24 ± 0.3 ^a^	15 ± 0.4	15.2 ± 0.7	15 ± 0.5
Increase, %	-	-	60	-	1.3	-
Control TOBRA	16 ± 0.0	16 ± 0.3	18 ± 0.0 ^b^	17 ± 0.7	17 ± 0.7	17 ± 0.7
Increase, %	-	-	12.5	6.3	6.3	6.3
***E. coli*** **, 27**						
Control GENT	15 ± 0.0	15 ± 0.0	21 ± 0.0 ^a^	17 ± 0.0 ^a^	15±0.0	15±0.0
Increase, %	-	-	40	13.3	-	-
Control AMI	17 ± 0.7	16.5 ± 0.3	31 ± 0.0 ^a^	22 ± 0.0 ^a^	17 ± 0.0	17 ± 0.0
Increase, %	-	-	82.4	29.4	-	-
Control TOBRA	15±0.7 ^a^	15 ± 0.0	27 ± 0.0 ^a^	19±0.0 ^a^	15 ± 0.0	15 ± 0.0
Increase, %	-	-	80	26.7	-	-
***B. cereus*** **, ATCC33018**						
Control GENT	18 ± 0.3	18.3 ± 0.0	23 ± 0.0 ^a^	18 ± 0.0	18 ± 0.0	18 ± 0.0
Increase, %	-	-	27.8	-	-	-
Control AMI	18 ± 0.0	18 ± 0.0	32 ± 0.0 ^a^	23 ± 0.0 ^a^	18 ± 0.0	18 ± 0.0
Increase, %	-	-	77.8	27.8	-	-
Control TOBRA	14 ± 0.0	14.5 ± 0.7	24 ± 0.0 ^a^	19 ± 0.3 ^a^	18 ± 0.0 ^a^	18 ± 0.0 ^a^
Increase, %	-	-	71.4	35.7	28.6	28.6
***S. aureus*** **, 358**						
Control GENT	22.0 ± 0.5	22.0 ± 0.5	26 ± 0.3 ^a^	24 ± 0.0 ^a^	22.0 ± 0.5	22.0 ± 0.5
Increase, %	-	-	18.2	9.1	-	-
Control AMI	25.0 ± 0.5	25.0 ± 0.5	35 ± 0.5 ^a^	27 ± 0.4 ^b^	25.0 ± 0.5	25.0 ± 0.5
Increase, %	-	-	40	8	-	-
Control TOBRA	25.5 ± 0.6	25.5 ± 0.6	34 ± 0.3 ^a^	30 ± 0.0 ^a^	25.5 ± 0.6	25.5 ± 0.6
Increase, %	-	-	33.3	17.6	-	-
***E. coli*** **, ATCC25922**						
Control GENT	17.0 ± 0.0	17.0 ± 0.0	20 ± 0.2 ^a^	19.2 ± 0.2 ^a^	17.0 ± 0.0	17.0 ± 0.0
Increase, %	-	-	17.6	12.9	-	-
Control AMI	20.5 ± 0.5	20.5 ± 0.5	21 ± 0.0	21 ± 0.0	20.5 ± 0.5	20.5 ± 0.5
Increase, %	-	-	2.4	2.4	-	-
Control TOBRA	17.0 ± 0.0	17.0 ± 0.0	30.0 ± 0.4 ^b^	28 ± 0.2 ^a^	17.0 ± 0.0	17.0 ±0.0
Increase, %	-	-	76.5	64.7	-	-

^a^ Abbreviations: AMI, amikacin; DMSO, dimethyl sulfoxide; GENT, gentamicin; N. TRA, no treated; EOHm, essential oil of *H. martiusii*; TOBRA, tobramycin.

^b^ Averages following by same letters, in the column, don't differ significantly amongst themselves (a) (n = 3, P < 0.05, test of Tukey). Averages following by different letters, in the line, differ significantly when compared with the respective control, for each microorganism (the n = 3, P < 0,001 ANOVA) and (b) (the n = 3, P < 0.01 ANOVA)

^c^ The results are expressed as Mean + SEM.

## 5. Discussion

A comparative study of the main components of the oil with those reported earlier showed variation even in the major chemical components reported for other *Hyptis* genus (23). In the work of Ferri and collaborators (29) were demonstrated chemical compositions of the essential oils of nine populations of *H. suaveolens* in fruiting stage from Brazilian Cerrado indicated the presence of 1.8-cineole (10.52%), sabinene (5.66%), limonene (7.06%), (E)-caryophyllene (11.96%), germacrene D (6.76%), bicyclogermacrene (11.39%), spathulenol (10.81%) and caryophyllene oxide (4.71). Chemical composition of *H. pectinata* essential oil indicated the presence of β-pinene (6.95%), β-caryophyllene (28.34%), caryophyllene oxide (28%), germacrene-D (3.07%) (30). The analysis of the essential oil *Hyptis ovalifolia* by GC/MS has enabled the identification of the following compounds: α-copaene (0.84%), β-bourbonene (1.58%), (Z)-caryophyllene (0.74%), γ-elemene (4.38%), α-humulene (1.05%), γ-cadinene (6.60%), viridiflorol (6.08%), caryophyllene oxide (4.98%) and α-cadinol (0.74%), among other minor constituents (25). In the diverse *Hyptis* species the presence caryophyllene, caryophyllene oxide are communes compounds but exist in different concentrations. These results suggest that geographical environmental factors influence the composition of the essential oil. Different studies have concluded that whole essential oils possess greater antibacterial activity than the mixed major components which suggests that the minor components might be critical to such activity, due to synergistic (or antagonism) effects. There is a current tendency not to consider MIC values above 200 mg/mL as promising; however, we disagree, for several reasons. Essential oil activity may be related to many other factors, such as oil volatility, water solubility, and general chemical complexity (31). The antimicrobial effect shown in this work reveals an important activity against bacteria present in several clinical diseases. The study of Asekun et al. (17) was reported that the oil of *H. suaveolens* show a significant inhibitory activity against gram-positive (*S. aureus* and *B. cereus*) and gram-negative (*E. coli* and *P. aeruginosa*) bacteria. The results the Nantitanon et al. (23) show that the essential oil of *H. suaveolens* inhibits the growth of different microorganisms, but, the oil is less active against gram-negative bacteria, particularly *P. aeruginosa* and *E. coli*, than gram-positive bacteria. This might be due to the protection by a hydrophilic outer membrane of the gram-negative bacteria which could suppress the passage of the lipophilic essential oil. Nascimento et al. (24) reported the antibacterial *Streptococcus mutans* activity of essential oil of *H. pectinata*, this activity was reported as major compounds of caryophyllene oxide (28%) and caryophyllene (28.3%) present in essential oil. Study of Santos et al. (30) show that essential oil of *H. pectinata* was most effective against Gram (+) bacteria and yeasts. This results was attributed, particularly the high percentage of β-caryophyllene, is a common feature of essential oils. de Melo et al. (15) highlight many studies which report on the antimicrobial properties of essential oils containing a significant sesquiterpene fraction. Santos et al. (30) demonstrated that the essential oil of *Hyptis pectinata* species also showed results with considerable antibacterial activity, especially in Gram-positive, where the highly pathogenic organisms such as *Staphylococcus aureus*, *Staphylococcus epidermidis*, *Bacillus subtilis* and *Enterococcus faecalis* were sensitive to the essential oil obtained from leaves *Hyptis* genus. Combining natural products with synthetic drugs to improve efficacy has also been investigated for other *Hyptis* genus (9, 18). Few works describe the modulatory activity aminoglycoside for gaseous contact; most of the researches uses the direct contact as form of determining the existence or not of synergism. Rodrigues (2009) (32) it accomplished research where she can observe satisfactory results when using the essential oil of *Croton zehntneri* Pax et Hoffm against bacterial lineages of *S. aureus* and *P. aeruginosa* ATCC15442. The activity of the gentamicin, one of the aminoglycoside also used in the research with *Hyptis martiusii*, it was modified. In the study the author used the essential oil of euphorbiaceae (*Croton zehntneri*) where verified an increment of antimicrobial effect against *P. aeruginosa* in 42.8% in the formation of the inhibition halo when the same was used together with the gentamicin. Sousa (33) was evaluated the modulatory antibacterial activity of the essential oil of *Lantana montevidensis* Briq. (Verbenaceae) for *Staphylococcus aureus* ATCC12692 and *Pseudomonas aeruginosa* ATCC 15442 by gaseous contact with aminoglycoside and show that inhibition of the growth of the both bacterial for gentamicin and amikacin antibiotics increased the inhibition halo 102%, demonstrating the synergistic effect of essential oil and antibiotics association. Different results were observed in antimicrobial modulation activity when comparison direct and gaseous contact, as observed in other studies (34). This difference can explain by the spontaneous degradation of unstable constituents by oxido-reduction reactions and rearrangement of linkage implying that they contributed significantly to the bioactivity. Kashiwagi et al. (35) was showed changes in 44 compounds of oil yuzu (*Citrus junos* Sieb) essential oil that accrue during storage at 25°C current of oxidative process. These study demonstration that Bicyclogermacrene, the main sesquiterpene hydrocarbon of the fresh oil, practically disappeared and convert spathulenol. Considering that caryophyllene oxide and bicyclogermacrene, that are present in EOHm oil, and spathulenol have been reported to present notable antibacterial activity against *S. aureus*. The presence of this substances in high yield in the oil obtained from the plants collected in the spring may be related to the antibacterial activity presented by that oil. This is the first report of the antimicrobial activity in vitro of the essential oil of *H. martiusii* against multidrug resistance bacteria by gaseous contact. The chemical constituents represent a real source of pharmacology, where each of the existing molecules may have activity either alone or jointly with others, which makes natural products potent biological agents. It is necessary to search for yet more substance and also by drug combinations that may respond satisfactorily, mainly bacterial resistance.

Antimicrobial action of essential oils by direct contact is less efficient when comparison with gaseous contact. This increase of activity will make it possible to formulate new oxygenated compounds when high vapor concentration of essential oil were exposed at air. The results obtained in this investigation suggested one possible clinical use of essential oil of *H. martiusii* may suppress the growth of the bacterial pathogens of respiratory infection. The data obtained from this work are promising and may stimulate further research on phytochemical, toxicological and pharmacological aspects of natural products isolated from the leaves of *Hyptis martiusii* Benth. In order to support their possible use in rational antimicrobial therapy and anti-multidrug resistance bacterial.
